# Expertise: defined, described, explained

**DOI:** 10.3389/fpsyg.2014.00186

**Published:** 2014-03-04

**Authors:** Lyle E. Bourne, James A. Kole, Alice F. Healy

**Affiliations:** ^1^Department of Psychology and Neuroscience, University of ColoradoBoulder, CO, USA; ^2^School of Psychological Sciences, University of Northern ColoradoGreeley, CO, USA

**Keywords:** expertise, definition, description, explanation, mind, brain

## Introduction

Science aims to define, describe, and explain significant natural phenomena. Each of these goals of science suggests an increasingly deeper understanding of the target phenomenon. We discuss in this paper how these goals are or might be realized in the science of expertise.

## Definition

Definitions are given in an attempt to identify phenomena and to delineate examples from non-examples. Expertise is consensually defined as elite, peak, or exceptionally high levels of performance on a particular task or within a given domain. One who achieves this status is called an *expert* or some related term, such as *virtuoso, master, maven, prodigy*, or *genius*. These terms are meant to label someone whose performance is at the top of the game. An expert's field of expertise can be almost anything from craftsmanship, through sports and music, to science or mathematics. People usually agree on examples of expertise, like Yo-Yo Ma (musical performance), Fred Astaire and Ginger Rogers (ballroom dancing), Antiques Roadshow Appraisers, Albert Einstein (physics), Tiger Woods (golf), Bette Davis (acting), Nelson Mandela (politics), or Hillary Rodham Clinton (international relations).

Why different terms? Each term carries with it a slightly nuanced meaning. Shaded meanings vary in their emphasis on experience or constitutional factors as the source of high levels of performance. The term chosen to characterize superior performance carries with it an implied cause. Like expert, *virtuoso* or *master* is the result of hard work and long training. If talent is involved, it is a talent for hard labor. In contrast, *prodigy*, like *genius*, results from an endowment, which shows up early in life without the benefit of training.

It might be appealing to the layperson to believe that a genius is just born that way. Elite performance just comes natural to a genius; you don't have to invest all that time and effort on training, because if you don't have what it takes you'll never get there. Moreover, you don't have to explain why you never had a significant insight, because you just didn't inherit the right abilities or genes. But the facts seem to be that, although people do differ in something called ability or talent, in sports or medicine or any area of human endeavor, talent is a necessary starting point, a platform from which to begin. To become an elite performer one has to capitalize on his or her abilities. Training is the *sine qua non*.

Consider a specific case. Pablo Picasso, Spanish painter and sculptor, was one of the greatest and most influential artists of the 20th century. Born into a family that cultivated the arts, he demonstrated extraordinary artistic ability at an early age, encouraged by his parents. All the elements were in place for Picasso—paints, brushes, canvases, and parents who could recognize good artistic work. Painting in the beginning in a naturalistic manner, his style changed later in life as he experimented with different theories, techniques, and ideas, for example, creating (with Braque) a unique style that has come to be known as cubism. There is no doubt that Picasso was a child *prodigy*. He had an ability to create significant objects that the art world and collectors recognized early on for their value. He seems to have been endowed with pure *genius* for painting and sculpting. But it is less often recognized that he was trained classically in the arts and that he worked incessantly at his craft, devoting long hours day and night. And, over time, the quality of his work improved, as judged by his peers, and expanded into previously unexplored areas and techniques. He could produce new paintings later in life quickly, some consisting of little more than three or four strokes of his pen, and more or less at will, each of them a *virtuoso* performance. But that performance was based on a level of *expertise* achieved, by dint of hard work, by few other mortals. Picasso is but one case of expertise and, as such, cannot validate a general rule. Nonetheless, his accomplishments are clearly based on a combination of ability and effort, a characteristic that other experts share.

## Description

We all know an expert when we see one. Normally people will quickly recognize the difference between expertise and normal or ordinary performance in any domain. Expertise, itself, is a descriptive term. To describe is to add detail in the specific case to a more general definition. A description of expertise requires an inventory of what the expert knows, knows how to do, wants or intends to do, and what he or she does or achieves. Psychologically, knowledge and skills are mental or cognitive concepts. They are not material entities, known by their physical make-up, but rather they are states of mind. This fact alone does not make them unscientific. Rather they are quite sound scientific concepts, known by their function, by the behavior potential they provide. Mind, knowledge, skill, and other cognitive concepts are analogous to gravity in physics or evolution in biology, understood in terms of their effects or functions, not by their material structure.

Obviously, there is more to expertise that just acquiring the right knowledge and skills. Expertise is based in some measure on the resources a person comes equipped with, his or her natural talent or biological endowment. We put an emphasis on practice and experience primarily because their contribution to expert performance is too often overlooked or minimized by the layperson (Ericsson et al., 1993). But clearly, inherited prodigiousness, body characteristics, dexterity, and the like, which are part and parcel of the equipment we come to any task with, all play a role, allowing some people just to be flat out better prepared than others. These natural factors provide essentially a foundation for expertise in any task. Given abilities and potential are useless unless they are capitalized on or activated by experience and practice, and, conversely, practice might be futile if one doesn't have some initial capacity. Both endowment and experience must be a part of a complete description of expertise.

Thus, to describe expertise is to identify the endowed resources, catalog the knowledge, and specify the skills of a person who is capable of performing in some domain at the very highest level, achieved by few others (perhaps by only a very small percentage of the general population).

## Explanation

How do experts get to be experts? What's the explanation? Is there something deeper than a description that we need to know about expertise? Maybe the brain or genes are at the bottom of it.

Mind and brain are often conflated terms and used interchangeably (Bourne and Healy, 2014). It is tempting to equate mind and brain, and it's quite commonly done among psychologists and other scientists, not to mention laypersons. Brain scanning is often used to study “how the mind works.” The general assumption behind brain-scanning procedures is that the brain provides a mechanism for mental functions. Thus, you commonly come across phrases such as, “How to train the brain,” “The brain learns (this, that, or the other thing),” “Learning is a rewiring of the brain,” or “The brain is the mind's machine.” The implication is that the brain causes thinking and behavior to be what they are. The psychological aspects of behavior are caused by a material, biological entity called the brain. Thus, the ultimate explanation for why and how we behave as we do is to be found in a material thing called the brain. In theories of this type, the brain is the *deus ex machine* that resolves difficulties we might have in understanding why people behave as they do.

But the facts of the matter are different. Training, experience, and practice directly change the mind (i.e., thought and behavior), but only indirectly the brain. It is a person or a mind, defined by the collection of all current knowledge and skill, that is trained, not a brain. A person learns, not the brain. That is not to say that the brain and what goes on in the brain are irrelevant, inconsequential, or unimportant in skill or knowledge acquisition. Quite the contrary, what happens in the brain as we learn and behave is essential to understanding the mind. As thinking happens, so do brain processes. Mind and brain processes are time-locked, and one can actually measure brain changes during thought. Still, there is no good reason to believe that one of these processes, say, brain activity, is more fundamental or causes the other, thought or behavior, to be what it is. In fact, the other causal direction—that thought causes brain activity to be what it is—is just as plausible. Consider the possibility that neither causes the other in a direct way but that both are going on in parallel simultaneously and in an interrelated way at all times. We think of that position as consistent with the long accepted first principle of the unity of the sciences. What we observe to be true in one domain of science should not conflict with what we observe at the same time in another domain of science. What we observe to be true psychologically should be consistent with what we observe biologically (or chemically, physically, etc.). Thus, mind (psychological) and brain (biological) are unique but different, and both will reflect, in their different ways, the expression of expertise in behavior.

So what is the explanation for expertise? Consider this, can you explain something you cannot first describe? Logically, we need to be able to describe expertise before attempting to explain it. That is why we tried to explicate description before attempting to deal with explanation. The more specific and detailed the description of a phenomenon, the better we understand it. So, given the right description of expertise, what are we missing? Reductionism asserts that explanations go beyond mere description to find more fundamental causes of the target behavior. The causes lie in more basic sciences. For psychological phenomena the immediate causes are likely to be biological. That's why the brain is often invoked as the controller, monitor, or generator of behavior.

Does brain activity then explain behavior? Does the explanation of expertise lie in brain circuitry or in genes? The correlations are there, between brain activity and behavior. But saying my brain made me do it is akin to saying “The Devil made me do it.” It's attributing a cause where there is no causal evidence. The available scientific evidence is strictly correlational. No one has yet demonstrated that the independent creation of a brain process will result in the specific behavior for which it is claimed to be the cause. Thus, asserting that the brain causes behavior is a matter of faith or belief. And faith has no place in science. Remember that correlation does not imply causation. Neither does correlation imply explanation. There is no good reason other than faith to believe that the explanation of behavior lies in biological events. The claim that psychological processes or behaviors cause biological events to be what they are is just as plausible or believable.

So, in our view, a scientific explanation (or “deep understanding”) of expertise, based on other sciences, remains to be realized. We suggest that, if thorough and complete descriptions of specific cases of expertise can be achieved, then there might be nothing left to explain, at least not in these cases. This possibility suggests that, among other things, the implied difference among the three goals of science (definition, description, explanation) is an illusion. Proper and complete description might supersede the need to explain.

But, if an explanation is to be sought, it will be found, in our view, in the domain of psychology, rather than some physical or biological science. By this psychological explanation, expertise results from practice and experience, built on a foundation of talent, or innate ability. The psychology laboratory has revealed empirically based training principles that further elucidate the explanation of expertise. These principles enable learners to maximize the acquisition, retention, and transfer of knowledge and skills, as summarized in Healy and Bourne (2012).

## Author contributions

Lyle E. Bourne Jr. wrote the initial and final drafts of the manuscript. James A. Kole and Alice F. Healy edited the initial draft of the manuscript and suggested revisions of it.
